# Identification of castration‐resistant prostate cancer‐related hub genes using weighted gene co‐expression network analysis

**DOI:** 10.1111/jcmm.15432

**Published:** 2020-06-02

**Authors:** Yifei Cheng, Lu Li, Zongshi Qin, Xiao Li, Feng Qi

**Affiliations:** ^1^ Department of Urologic Surgery Jiangsu Cancer Hospital & Jiangsu Institute of Cancer Research & Affiliated Cancer Hospital of Nanjing Medical University Nanjing China; ^2^ Department of Urology The First Affiliated Hospital of Nanjing Medical University Nanjing China; ^3^ Nanjing Medical University Nanjing China; ^4^ School of Chinese Medicine Li Ka Shing Faculty of Medicine The University of Hong Kong Hong Kong China

**Keywords:** CRPC, hub genes, prognosis, WGCNA

## Abstract

Prostate cancer is the most common malignancy in urinary system and brings heavy burdens in men. We downloaded gene expression profile of mRNA and related clinical data of GSE70768 data set from public database. Weighted gene co‐expression network analysis (WGCNA) was used to identify the relationships between gene modules and clinical features, as well as the candidate genes. Kyoto Encyclopedia of Genes and Genomes (KEGG) and Gene Ontology (GO) analyses were developed to investigate the potential functions of related hub genes. Importantly, basic experiments were performed to verify the relationship between hub genes and the phenotype previously identified. Lastly, copy number variation (CNV) analysis was conducted to explore the genetical alteration. WGCNA identified that black module was the most relevant module which was tightly related to castration‐resistant prostate cancer (CRPC) phenotype. KEGG and GO analysis results revealed genes in black module were mainly related to RNA splicing. Additionally, 9 genes were chosen as hub genes and heterogeneous nuclear ribonucleoprotein A2/B1 (*HNRNPA2B1*), golgin A8 family member B (*GOLGA8B)* and mitogen‐activated protein kinase 8 interacting protein 3 (*MAPK8IP3*) were identified to be associated with PCa progression and prognosis. Moreover, all above three genes were highly expressed in CRPC‐like cells and their suppression led to hindered cell proliferation in vitro. Finally, CNV analysis found that amplification was the main type of alteration of the 3 hub genes. Our study found that *HNRNPA2B1*, *GOLGA8B* and *MAPK8IP3* were identified to be tightly associated with tumour progression and prognosis, and further researches are needed before clinical application.

## 1 INTRODUCTION

Prostate cancer (PCa) is the most common malignancy in urinary system, leading to the fifth cause of death in male worldwide. In 2018, the global estimated new cases and deaths were 1 276 106 and 358 989, respectively.^1^ Localized PCa patients can be treated effectively via various methods including active surveillance, watchful waiting, surgery and radiation. However, management for advanced diseases remains to be a difficult problem for urologists. Generally, androgen deprivation therapy (ADT), raised by Charles Huggins ^2^ firstly in the 1940s, is the standard treatment for advanced PCa patients. Types of ADT forms mainly include medical castration (luteinizing hormone‐releasing hormone (LHRH) agonist) and surgical castration (bilateral orchiectomy). Unfortunately, ADT therapy for these castration‐resistant prostate cancer (CRPC) patients is not curative. As a result, progressing to CRPC condition is inevitable within two years.^3^, ^4^, ^5^

Taken together, previous data indicated that 10%‐20% of all PCa patients develop CRPC within approximately 5 years from initial diagnosis. More than 84% of CRPC patients had metastases at diagnosis with bone as the most preferential site.^6^ For those who had no metastases at diagnosis of CRPC, 33% of patients were estimated to develop metastases within 2 years.^7^ Radionuclide therapy, radiotherapy, bisphosphonates and opioids were the most common treatment types for those patients with metastatic CRPC and skeletal symptoms. A systematic review showed that only about one‐third of CRPC patients received chemotherapy while the rest could only receive supportive care or steroids.^8^ Prognosis of CRPC patients is poor, especially for those patients with metastatic CRPC. Study conducted by Emmanuel S. Antonarakis et al showed that the median survival from CRPC initial diagnosis was 14 months while the overall survival of patients with metastatic CRPC was only 9‐13 months.^8^, ^9^

Nowadays, the specific mechanisms underlying the development of castration resistance have not been well discussed and strategies of CRPC treatment are limited. Study conducted by Wu et al^10^ showed that PCa patients with variant allele of *HSD3B1* increased the risk of progressing to CRPC, but it was not associated with biochemical recurrence, mortality or overall survival. Joshua W. Russo et al^11^ proposed that down‐regulation of dipeptidyl peptidase 4 promoted progression to CRPC. With the development of DNA microarray and high‐throughput sequencing, it is convenient to explore hub genes related to tumour process or progression benefited from bioinformatics techniques and big data integration. Weighted gene co‐expression network analysis (WGCNA) is a powerful biological method which can systematically investigate highly synergistically altered gene modules. It can thoroughly study the relationship between gene sets and clinical characteristics, and identify possible biomarkers for further researches. In this study, we aimed to find and validate differentially expressed genes (DEGs) which were associated with castration resistance in PCa by WGCNA. In addition, Kyoto Encyclopedia of Genes and Genomes (KEGG) and Gene Ontology (GO) analyses were developed to investigate the potential functions of hub genes in the key module.

## 2 METHODS AND MATERIALS

### 2.1 Data processing

**FIGURE 1:**
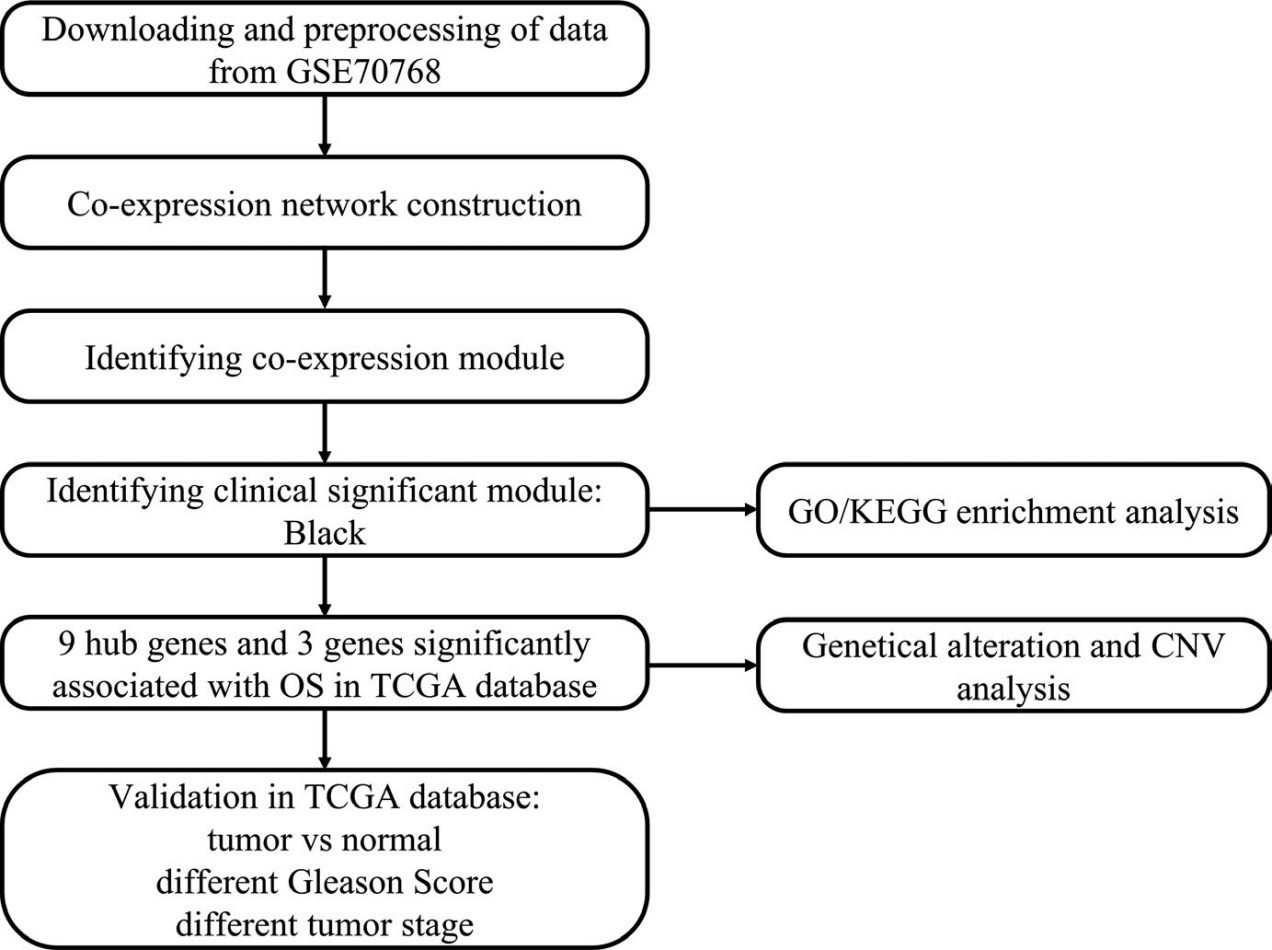
Flow chart of data preparation, processing, analysis and validation

A flow chart of this study was shown in Figure 1. Gene expression profile of mRNA and related clinical data of PCa patients were downloaded from Gene Expression Omnibus (GEO) database (http://www.ncbi.nlm.nih.gov/geo/). GSE70768 included 16 331 genes and 125 tumour tissues, which was used to construct WGCNA for this study. Among them, 13 were CRPC tissues and 112 were hormone‐sensitive prostate cancer (HSPC) tissues. The Cancer Genome Atlas (TCGA) data portal (https://portal.gdc.cancer.gov/) was used for validation of hub genes. The data of mRNA expression were transformed to values in transcripts per million (TPM).

### 2.2 Construction of the co‐expression network

‘WGCNA’ R package was applied to find clinical traits‐related modules and hub genes.^12^ The formulas used to calculate the interaction coefficient between genes and transform the connection coefficient into a weighted coefficient were specifically described in previous studies.^13^ The top 5000 most variant genes by analysis of variance were retained for WGCNA analysis.

Firstly, we use pickSoftThreshold function to find a soft threshold power β in accordance with standard scale‐free networks. Second, the adjacencies between all filtered genes were calculated using the power adjacent function to Pearson's correlation matrix to transform data into a topological overlap matrix (TOM), and the corresponding dissimilarity (1‐TOM) was calculated. Then, the dissimilarity of module eigengenes was calculated to further analyse the module. Finally, we set soft‐thresholding power of 9, cut‐off height of 0.25 and minimal module size of 30 to identify key modules.

### 2.3 Identification of clinically significant modules

The module eigengene (ME) was defined as the first principal component of a given module, which could be regarded as a representative of the gene expression profiles from a module. Gene significance (GS) was defined as the log10 transformation of the *P* value (GS = lgP) in the linear regression between gene expression and pathological progression. Module significance (MS) represented the average GS for all the genes in a module. In a word, the module with the absolute MS ranked first among all the selected modules was chosen as the one related with clinical trait.

### 2.4 Pathway enrichment and GO analysis

DAVID (http://david.abcc.ncifcrf.gov/) is a database for annotation, visualization and integrated discovery. KEGG pathway and GO analysis of genes in the module with the highest correlation with clinical traits were carried out using DAVID (version 6.8) online tools: functional annotation. Enriched GO terms and KEGG pathways were identified according to the cut‐off criterion of *P* < 0.1.

### 2.5 Hub genes detection and validation

Hub genes were defined as those with cor.geneTraitSignificance > 0.2 and cor.geneModuleMembership > 0.85. Then, TCGA‐PRAD data were applied to perform survival analysis to validate and select genes with clinical significance. Then, we compared the expression of genes between tumours and normal tissues along with expression between tumours with different clinical status in TCGA database.

### 2.6 Cell culture and transfection

The human androgen‐dependent PCa cell line LNCaP (ATCC, Manassas, VA, USA) and three CRPC‐like cell lines including LNCaP‐AI, LNCaP‐Bic and C4‐2 (ATCC) were applied in this study. LNCaP and C4‐2 cells were maintained in RPMI‐1640 medium (Gibco, Waltham, MA, USA) containing 10% foetal bovine serum (FBS; Gibco) and 1% penicillin/streptomycin (Gibco). LNCaP‐AI cells were grown in RPMI‐1640 without phenol red and supplemented with 10% charcoal‐stripped FBS (Gibco), whereas LNCaP‐Bic cells were added with 20 μM bicalutamide (Sigma, St. Louis, MO USA). Prior to any experiment, LNCaP cells were maintained in phenol red‐free medium containing charcoal‐stripped FBS for no less than 48 hours. All cells were incubated under a humidified atmosphere with 5% CO_2_ at 37°C.

With respect to cell transfection, LNCaP‐AI and C4‐2 cells were transfected with indicated short hairpin RNAs (shRNAs), such as sh‐HNRNPA2B1#1/2, sh‐GOLGA8B#1/2, sh‐MAPK8IP3#1/2 and corresponding control sh‐NC, by use of Lipofectamine 2000 (Invitrogen, Waltham, MA, USA) in line with manufacturers’ guide.

### 2.7 Cell proliferation detection

Cell proliferation was analysed via colony formation and EdU (5‐ethynyl‐2’‐deoxyuridine) assays. For Colony formation assay, 1 × 10^3^ cells were seeded into six‐well plates with three replicates. Two weeks later, colonies were stained via 0.5% crystal violet and those with more than 50 cells were counted after imaged. For EdU assay, EdU staining kit (Ribobio) was used based on the protocol. In brief, cells in 96‐well plates were processed with 50 μmol/L EdU medium (100 μL) for 3 hours. After being fixed and permeabilized, cells were stained via DAPI and then observed and imaged under a fluorescence microscope (Olympus, Tokyo, Japan).

### 2.8 Cell apoptosis analysis

Cell apoptosis was examined by TUNEL (terminal dexynucleotidyl transferase‐mediated dUTP nick end labelling) assay and flow cytometry analysis. In regard to TUNEL assay, cells were permeated with 0.1% Triton X‐100, rinsed in phosphate buffer saline (PBS) and then treated with TUNEL reaction for 1 hour according to the protocol of the TUNEL staining kit. Finally, apoptotic cells were observed and pictured by Olympus fluorescence microscope. As for flow cytometry analysis, cells were treated with 15 minutes of staining of FITC‐Annexin V and propidium iodide (PI), followed by analysis via flow cytometer (BD Biosciences, Franklin Lakes, NJ, USA).

### 2.9 Genetical alteration of hub genes and protein‐protein interaction (PPI) analysis

The cBioPortal for Cancer Genomics (http://www.cbioportal.org/) is a large‐scale cancer genomics database. We select four data sets associated with metastasis (SU2C, MICH, SU2C219 and MPC Project) to explore genetic alterations connected with the 9 hub genes. Next, copy number variation (CNV) data from TCGA database were used to conduct CNV analysis. Samples were classified into three groups: amplification, wild‐type and deletion according to the CNV data, and the expression levels of genes were compared among these groups. Finally, we applied String (https://string‐db.org/) and cystoscope (version 3.7.1) to construct protein‐protein interaction network of hub genes.

### 2.10 Statistical analyses

Kaplan‐Meier curves and the log‐rank tests were used to assess association between mRNA expression and overall survival of PCa patients. Relationships between mRNA expression and clinical or CNV features were assessed by the Wilcoxon rank‐sum test for comparison between two groups and ANOVA for comparison between more than two groups. All statistical analyses were performed using R (version 3.6.1). All statistical tests were 2‐sided, and *P* < 0.05 was considered statistically significant.

## 3 RESULTS

### 3.1 Weighted co‐expression network and Key Modules

**FIGURE 2:**
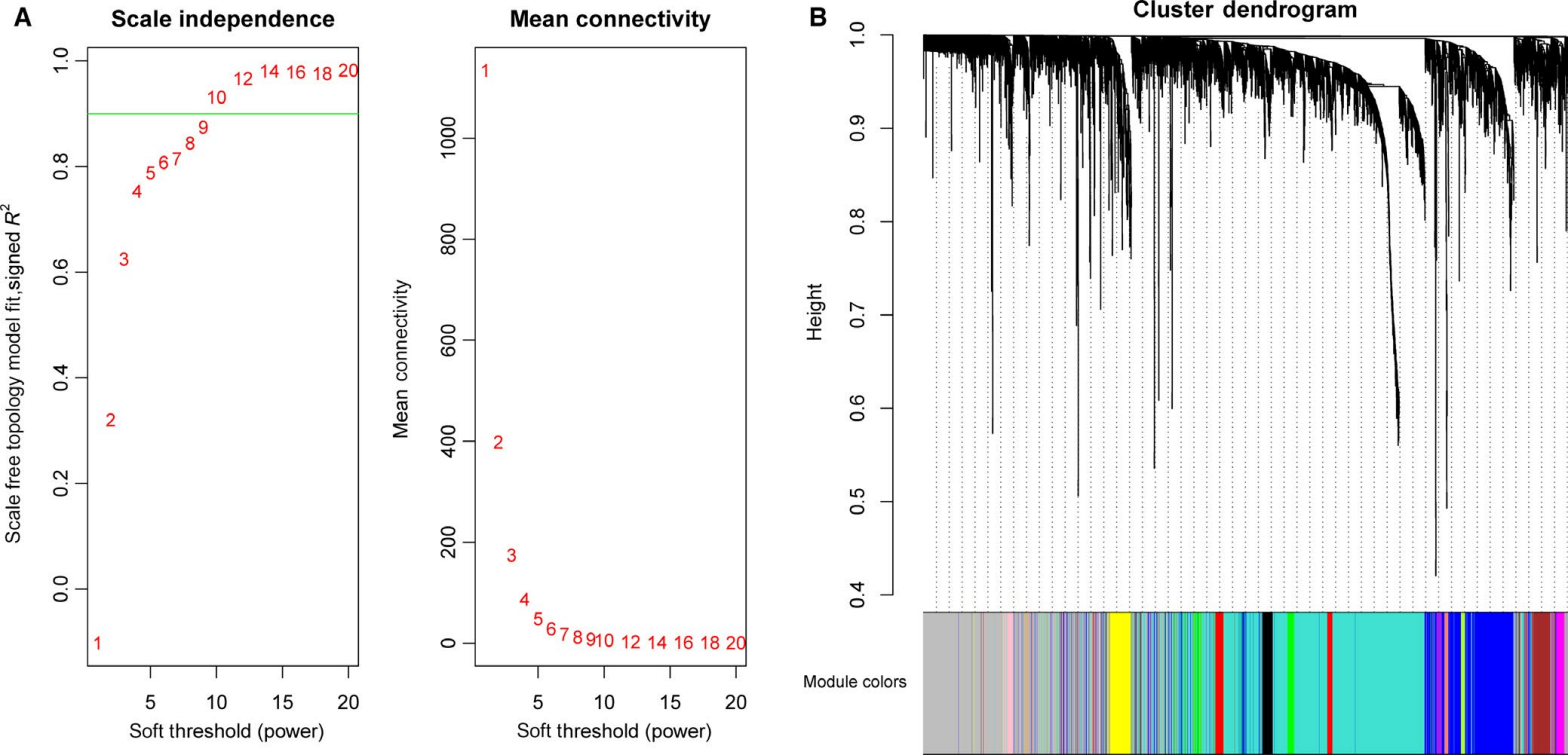
Determination of soft‐thresholding power in the WGCNA and identification of modules. A, Analysis of the scale‐free fit index for various soft‐thresholding powers (left). Analysis of the mean connectivity for various soft‐thresholding powers (right). B, Dendrogram of all differentially expressed genes was clustered based on a dissimilarity measure (1‐TOM). 13 modules were identified

**FIGURE 3:**
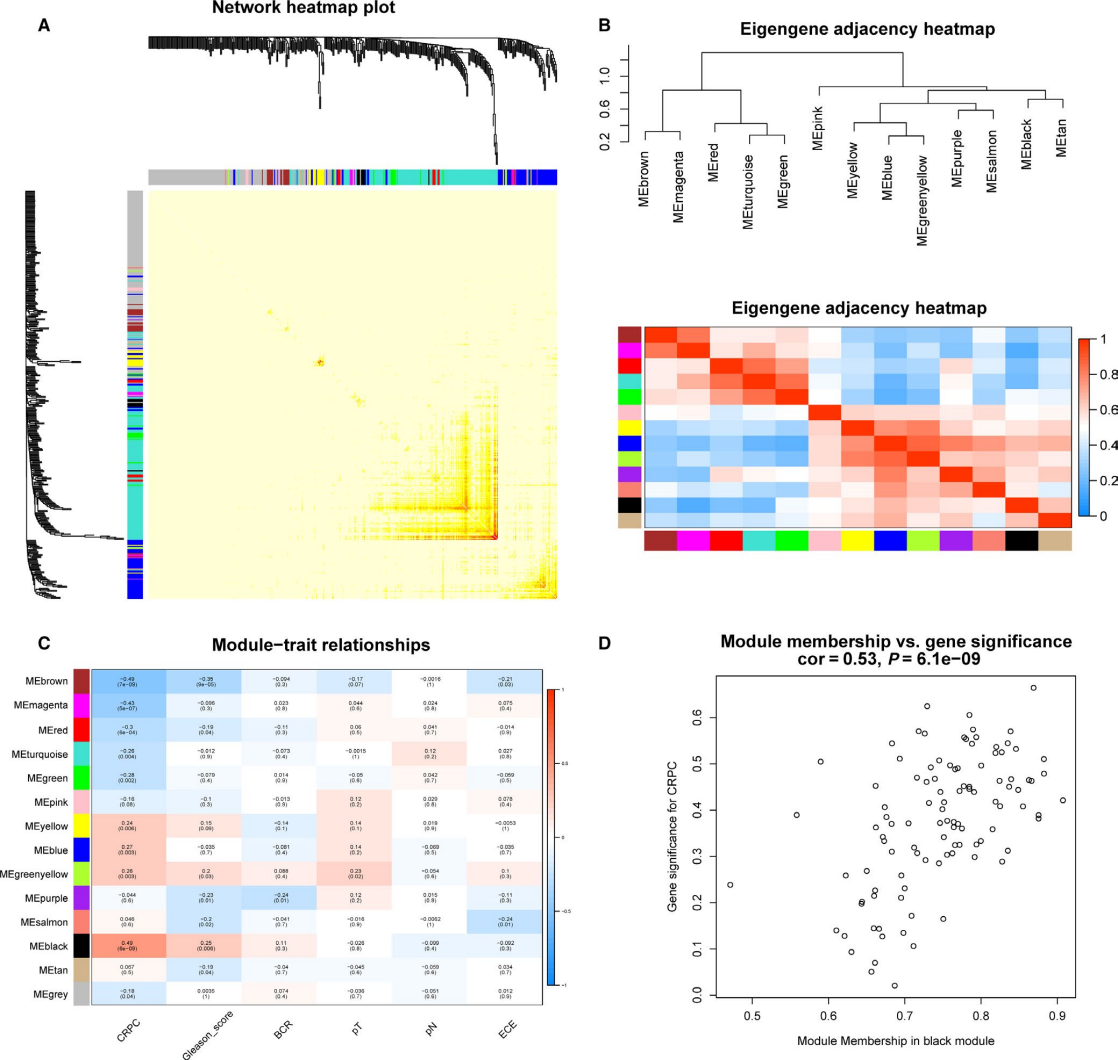
Module interaction and identification of modules associated with the clinical traits of PCa. A, The heatmaps of the correlations between 13 modules. B, Clustering of modules eigengenes and heatmaps of the correlations between eigengenes. C, Heatmap of the correlation between module eigengenes and clinical traits of PCa. D, Scatter plot of module eigengenes in the black module

Pearson's correlation method and average linkage method were used to cluster the samples from GSE70768 data set (Figure S1). Then, co‐expression network was constructed using co‐expression analysis. Under the soft‐thresholding power of 9, cut height of 0.25 and minimal module size as 30, we identified 13 modules (Figure 2). Later, the heatmaps of the correlations confirmed the independence of these 13 modules and gene expression in each module (Figure 3A‐B). After module‐trait relationship analysis, we observed that black module had the highest association with castration resistance (Figure 3C). In addition, a strong relationship was detected between genes in black module and CRPC (*P* = 6.1 × 10^−9^, Figure 3D). Consequently, we focused on black module and the clinical characteristic of CRPC in our further study.

### 3.2 Pathway enrichment and gene ontology analysis

**FIGURE 4:**
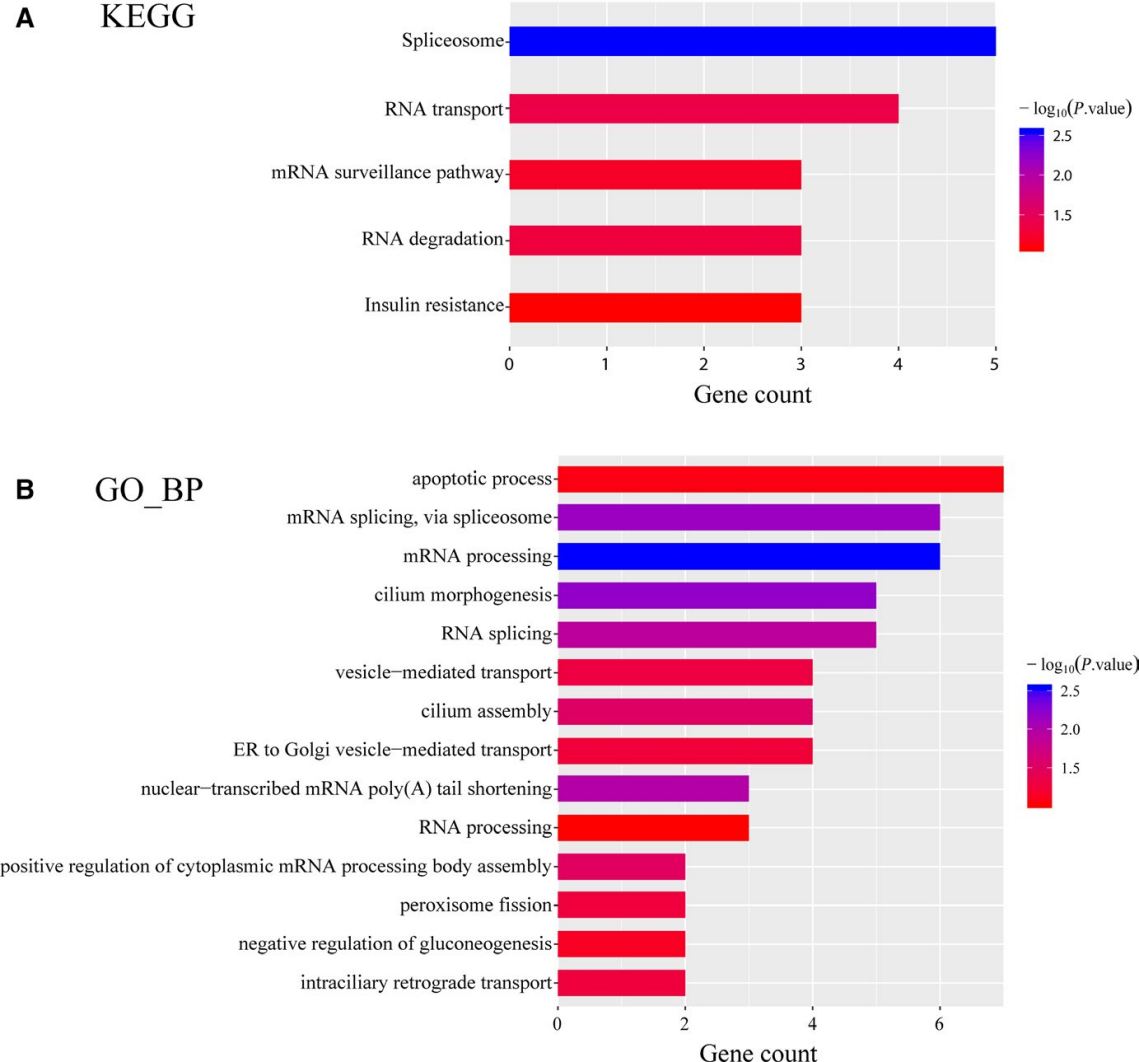
KEGG pathway and GO analysis of hub genes in black module. A, Bar chart of KEGG pathway analysis for genes in the black module. B, Bar chart of biological process of GO analysis for genes in the black module. KEGG, Kyoto Encyclopedia of Genes and Genomes; GO, Gene Ontology

KEGG pathway enrichment and GO analysis were applied to perform gene annotation. Figure 4A exhibited the result of KEGG pathway enrichment. The most significant pathway was spliceosome pathway and the other significant pathways included RNA transport, surveillance and degradation pathways. Biological process (BP) of GO analysis showed genes in the black module were mainly associated with RNA splicing, mRNA processing, cilium morphogenesis and apoptotic process (Figure 4B). In a word, the black module genes were mainly related to the regulation on RNA level involving RNA splicing, processing, transport, surveillance and degradation.

### 3.3 Hub genes identification and validation

**FIGURE 5:**
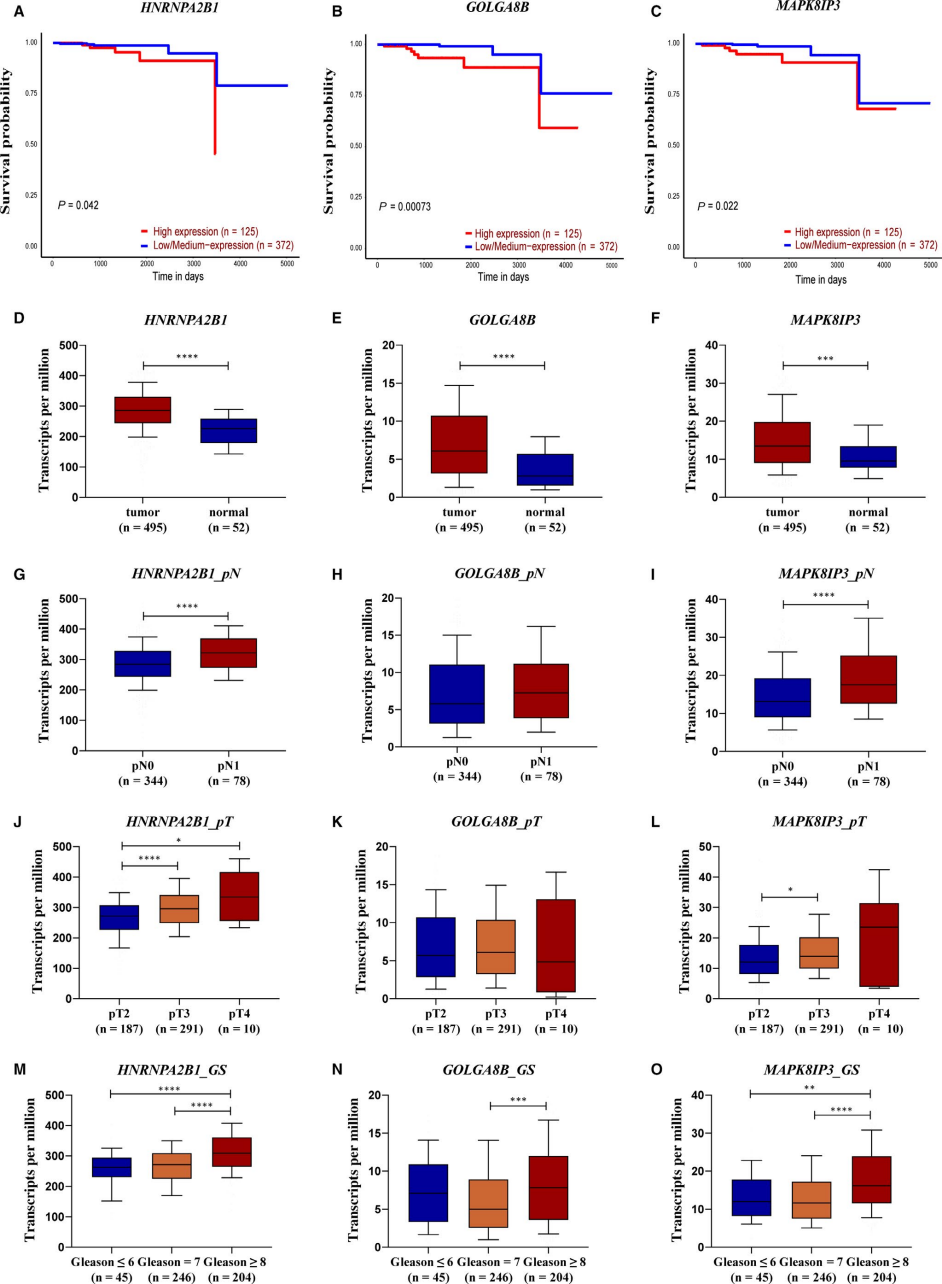
Validation of hub genes in black module. A‐C, Association between HNRNPA2B1, GOLGA8B and MAPK8IP3 expression and overall survival time in the TCGA‐PRAD data set. D‐F, Expression difference between tumour and normal tissues of HNRNPA2B1, GOLGA8B and MAPK8IP3 in the TCGA‐PRAD data set. G‐I, Expression difference between tumour tissues with different pathological N stages of HNRNPA2B1, GOLGA8B and MAPK8IP3 in the TCGA‐PRAD data set. J‐L, Expression difference between tumour tissues with different pathological T stages of HNRNPA2B1, GOLGA8B and MAPK8IP3 in the TCGA‐PRAD data set. (M‐O) Expression difference between tumour tissues with Gleason score of ≤6, =7 and ≥8 of HNRNPA2B1, GOLGA8B and MAPK8IP3 in the TCGA‐PRAD data set

Nine genes with the high connectivity in black module were chosen as hub genes based on the criteria that cor.geneModuleMembership > 0.85 and cor.geneTraitSignificance > 0.2. Among them, heterogeneous nuclear ribonucleoprotein A2/B1 (*HNRNPA2B1*), golgin A8 family member B (*GOLGA8B*) and mitogen‐activated protein kinase 8 interacting protein 3 (*MAPK8IP3*) were found significantly associated with overall survival of PCa patients in TCGA database (*P* = 4.2 × 10^−2^, 7.3 × 10^−4^ and 2.2 × 10^−2^ for *HNRNPA2B1*, *GOLGA8B* and *MAPK8IP3*, respectively, Figure 5A‐C). The Kaplan‐Meier Plotter of the rest 6 genes was shown in Figure S2.

**FIGURE 6:**
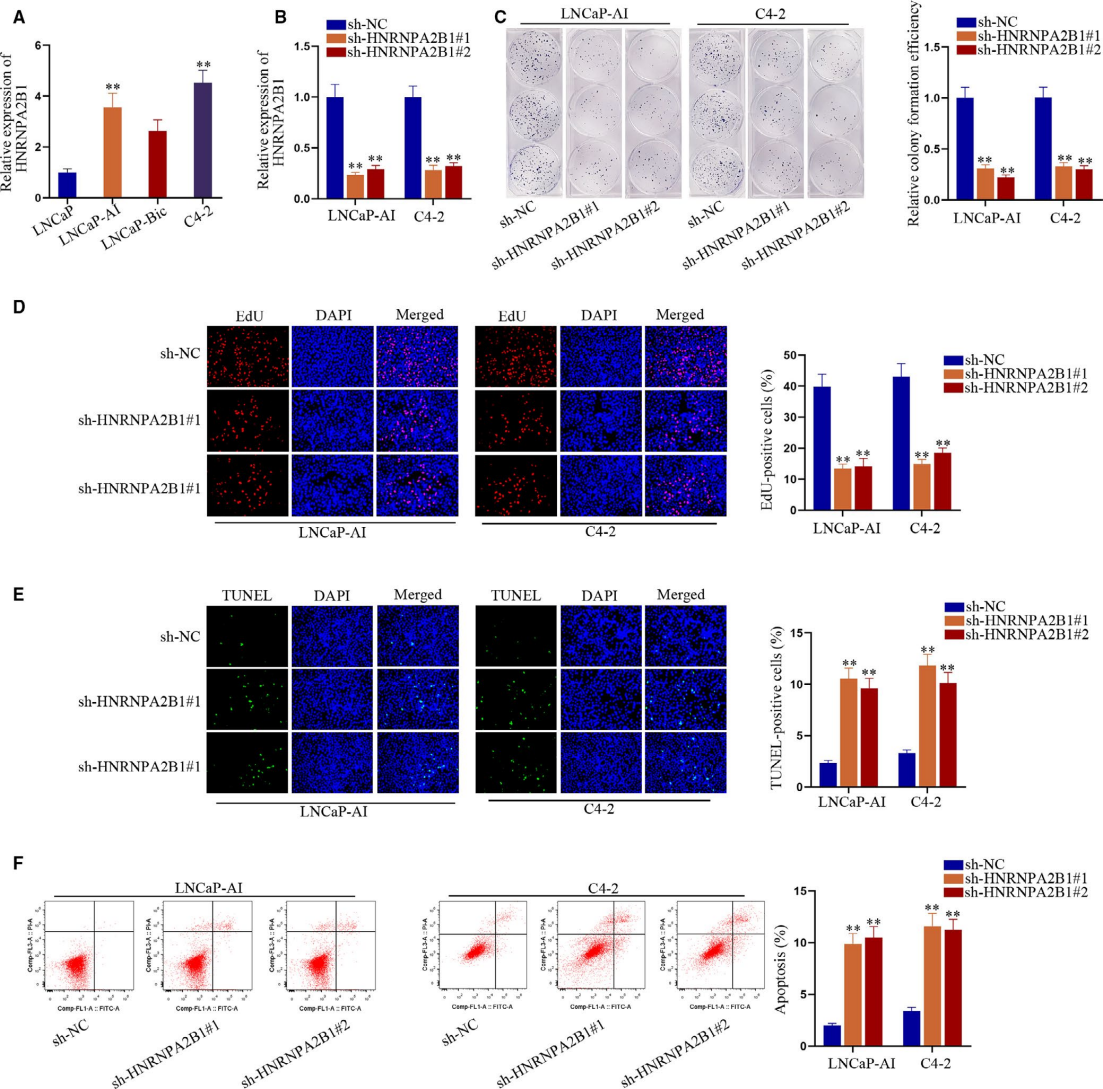
Silencing HNRNPA2B1 impaired CRPC cell proliferation and accelerated cell apoptosis in vitro. A, Expression trend of HNRNPA2B1 in CRPC‐like cells compared to androgen‐dependent LNCaP cells. B, Knockdown efficiencies of sh‐HNRNPA2B1#1/2 in LNCaP‐AI and C4‐2 cells. C‐D, Colony formation and EdU assays were carried out to evaluate cell proliferation under HNRNPA2B1 silence. E‐F, Cell apoptosis in two CRPC‐like cells with or without HNRNPA2B1 suppression was detected via TUNEL assay and flow cytometry analysis. **P < 0.01

We further evaluated the relationship between these 3 genes and PCa risk. As shown in Figure 5D‐F, dramatic differences were found in the expression of these 3 genes between tumours and normal tissues (*P* = 3.0 × 10‐11, 9.4 × 10^−7^ and 4.1 × 10^−4^ for *HNRNPA2B1*, *GOLGA8B* and *MAPK8IP3*, respectively). In addition, high expression of *HNRNPA2B1* and *MAPK8IP3* was significantly associated with worse pathological N and T stage (*P* = 5.3 × 10^−5^ and 3.6 × 10^−5^ for *HNRNPA2B1* and *MAPK8IP3* in pN1 vs pN0, respectively. *P* = 4.1 × 10^−5^ and 2.1 × 10^−2^ for *HNRNPA2B1* in pT3 vs pT2 and pT4 vs pT2, respectively. *P* = 1.2 × 10^−2^ for *MAPK8IP3* in pT3 vs pT2. Figure 5G‐L). Besides, significant differences between high‐risk PCa patients [Gleason score (GS) ≥ 8] and low‐ or medium‐risk PCa patients (GS ≤ 6 or =7) were detected in all these 3 genes (*P* = 8.5 × 10^−6^ and 5.0 × 10^−3^ for *HNRNPA2B1* and *MAPK8IP3* in GS ≥ 8 vs GS ≤ 6, respectively. *P* = 2.3 × 10^−10^, 1.8 × 10^−4^ and 2.2 × 10^−10^ for *HNRNPA2B1*, *GOLGA8B* and *MAPK8IP3* in GS ≥ 8 vs GS = 7, respectively. Figure 5M‐O). More importantly, we discovered that *HNRNPA2B1* was highly expressed in CRPC‐like cell lines and inhibition of *HNRNPA2B1* could hamper CRPC cell proliferation but facilitate apoptosis (Figure 6). As for the expression pattern and function of *GOLGA8B* and *MAPK8IP3* in CRPC‐like cells, we observed similar phenomena as that of *HNRNPA2B1* (Figure S3 and S4). In one sentence, *HNRNPA2B1, GOLGA8B* and *MAPK8IP3* might be significant for tumorigenicity of CRPC.

### 3.4 Genetical alteration of hub genes and CNV analysis

**FIGURE 7:**
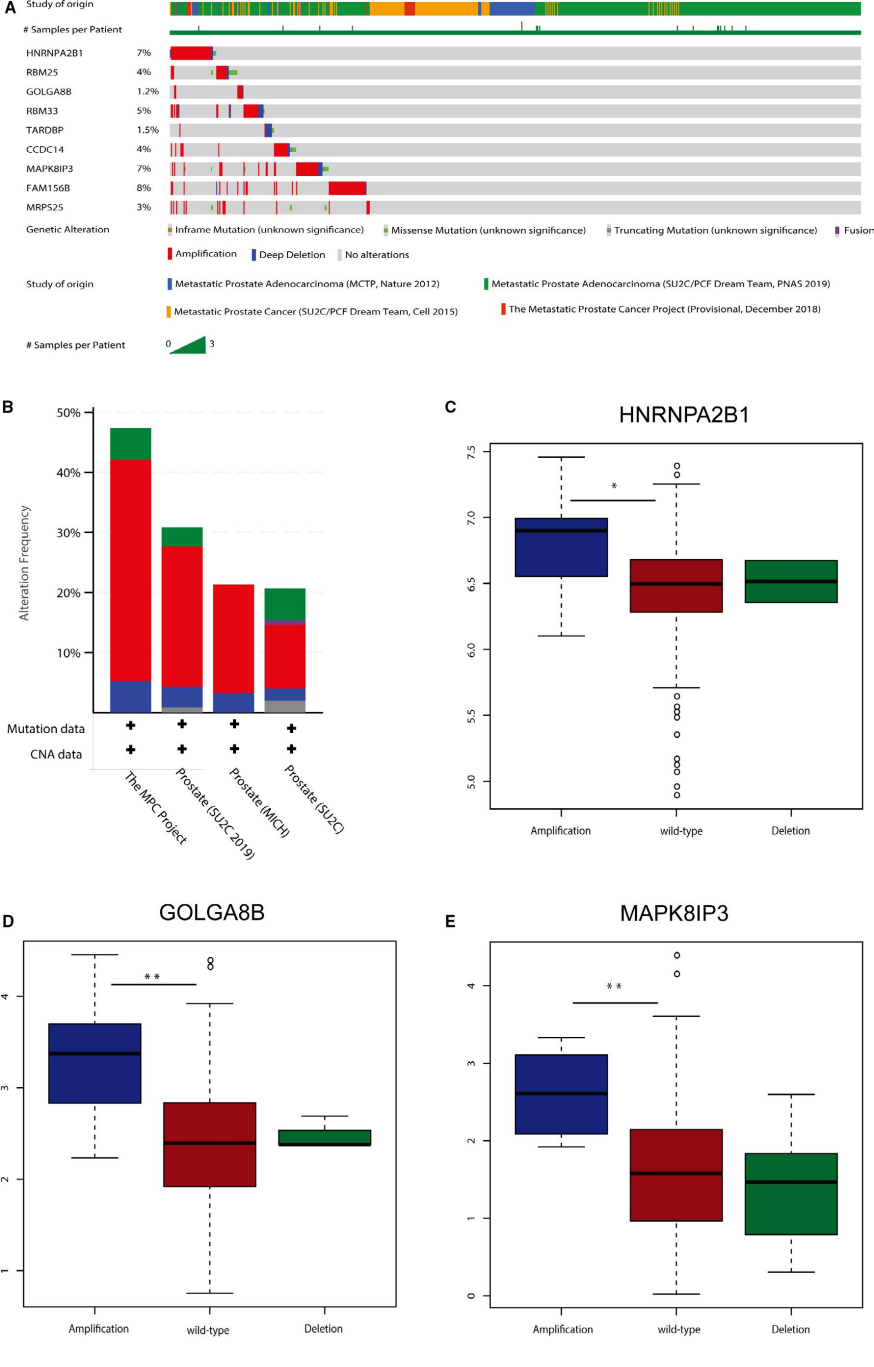
Genetic alterations associated with 9 hub genes. A, A visual summary of Genetic alterations (data from SU2C, MICH, SU2C219 and MPC Project) shows the genetic alteration of 9 hub genes. B, The total alteration frequency of 9 hub genes is illustrated. C‐E, Expression difference among amplification, wild‐type and deletion groups of HNRNPA2B1, GOLGA8B and MAPK8IP3 in the TCGA‐PRAD data set

The genetical alteration of the 9 hub genes was analysed using data concerning metastasis from cBioPortal database. The alteration frequency of each hub gene was shown in Figure 7A. It was shown that FAM156B, *HNRNPA2B1* and *MAPK8IP3* altered most (8%, 7% and 7%, respectively, Figure 7A). In addition, we found that amplification was the main type of alteration in all the four subjects (Figure 7B). The CNV analysis of gene *HNRNPA2B1*, *GOLGA8B* and *MAPK8IP3* confirmed the result that there existed abundant of amplification among this group of genes (Figure 7C‐E). Figure S5 showed the relationship of the genes in the black module and the other frequently altered neighbour genes. *HNRNPA2B1* and RBM25 were significantly important in the network.

## 4 DISCUSSION

PCa is a heterogeneous disease, and its pathogenesis remains unclear. Many studies have explored novel biomarkers that related to oncogenesis, tumour progression and prognosis using RNA sequencing and gene chip. However, huge inconsistencies existed between the DEGs found in these studies.^14^ Song et al^15^ discovered *LMNB1*, *TK1*, *RACGAP1* and *ZWINT* as candidate genes for diagnosis and prognosis of PCa using Robust Rank Aggregation (RRA) method. Hou et al^16^ developed a prognostic and diagnostic prediction model for PCa, and proposed *C1QTNF3* as a promising gene for PCa utilizing RankProd method.

The nine hub genes selected in this study include *HNRNPA2B1*, *GOLGA8B*, *MAPK8IP3*, *CCDC14*, *MRPS25*, *RBM25*, *RBM33*, *TARDBP* and *FAM156B*. All these hub genes were oncogenes and tightly related to the clinical characteristics of CRPC. Most of these key genes were identified as important biomarkers in some other diseases, such as breast cancer,^17^ lung cancer,^18^ pancreatic ductal adenocarcinoma,^19^ clear cell renal cell carcinoma^20^ and so on. Eventually, *HNRNPA2B1*, *GOLGA8B* and *MAPK8IP3* were picked out to be significantly associated with tumour prognosis. In order to further investigate potential mechanism of 9 hub genes, KEGG pathway analyses showed that genes in black module were mainly enriched in spliceosome pathway, RNA transport, surveillance and degradation pathways. Furthermore, GO analysis revealed that the hub genes were mainly associated with RNA splicing, mRNA processing, cilium morphogenesis and apoptotic process. Among the significantly enriched pathways, RNA transport and spliceosome pathway have been proven to be associated with tumour progression and prognosis in some cancer types, such as colorectal cancer,^21^ hepatocellular carcinoma,^22^ oesophageal squamous cell carcinoma^23^ and so on.

Heterogeneous nuclear ribonucleoproteins (hnRNPs) are RNA‐binding proteins which are expressed in most human tissues. They stick to nascent transcript and are mainly involved in DNA repair, messenger RNA (mRNA) biogenesis, gene expression regulation, cell signalling and telomere biogenesis.^24^ Previous studies have already investigated the prognostic role of *HNRNPA2B1* in many cancer types. Zhou et al^25^ discovered that *HNRNPA2B1* was over‐expressed in breast cancers, and the same conclusion could be drawn in lung cancers.^18^, ^26^ Deng et al demonstrated that *HNRNPA2B1* was a driver oncogene in glioblastoma and acted as a predictor of overall survival.^27^ Carles Barceló et al found that *HNRNPA2B1* interacted with oncogenic *KRAS* in pancreatic ductal adenocarcinoma cells.^19^ *GOLGA8B* is a protein‐coding gene, which is supposed to be an important factor in the Golgi structure maintenance. The association between *GOLGA8B* and tumour progression or prognosis has not been explored deeply. The only corresponding study was reported by Wang et al, they found that over‐expression of *GOLGA8B* might result in poor overall survival and *GOLGA8B* was a potential therapeutic target in patients with clear cell renal cell carcinoma.^20^ *MAPK8IP3*, also known as c‐Junamino‐terminal kinase (*JNK*)/stress‐activated protein kinase‐associated protein 1 (*JSAP1*), was originally thought to be a binding protein for JNK and was suggested as a scaffold protein in neuronal cells via mammalian JNK signalling pathways.^28^, ^29^ Furthermore, many studies have suggested the important role of *JSAP1* in certain neuronal processes, including axon elongation and branching.^30^, ^31^, ^32^

Mammalian mitochondrial ribosomal proteins are encoded by nuclear genes and are involved in the protein synthesis within the mitochondrion. Mitochondrial ribosomes (mitoribosomes) consist of a small 28S subunit and a large 39S subunit. *MRPS25*, one of 15 structural subunits of the 28S subunit, does not have a bacterial homolog.^33^ Role of *MRPS25* has not been explored in CRPC previously. Study conducted by Enrico Bugiardini et al showed that *MRPS25* mutations could impair mitochondrial translation and cause encephalomyopathy.^34^ *RBM25* and *RBM33* are family memberships of RNA‐binding proteins (RBPs), which are involved in the co‐ and post‐transcriptional process, influencing mRNA biogenesis, transport, stability, modification and cellular localization. However, rare research has been made on the role of *RBM25* and *RBM33*. In this study, over‐expression of *RBM 25* and *RBM 33* was closely related to CRPC phenotype. *FAM156B*, also known as *TMEM29B*, is a protein‐coding gene with ubiquitous expression in skin, placenta, spleen, fat, adrenal and so on. Zané Lombard et al^35^ found that *FAM156B* was a candidate gene utilizing a computational approach. Activation of HIV‐1 gene expression by the transactivator Tat is dependent on an RNA regulatory element (TAR) located downstream of the transcription initiation site. *TARDBP*, a TAR DNA binding protein, is ubiquitous expressed, especially in lymph node and appendix. Protein encoded by *TARDBP* is a transcriptional repressor which binds to integrated TAR DNA chromosomally and represses HIV‐1 transcription. There are few references on the role of *CCDC14* on transcriptome level. Huang et al^36^ identified *CCDC14* as a candidate gene associated with autism spectrum disorder. Elif Nur Firat‐Karalar et al reported that *CCDC14* played an important role in the interactions among centrosome components.^37^

Recently, many previous studies were developed to identify key genes in CRPC by integrated bioinformatics methods. Wang et al^38^ discovered PTPRR and JAG1 as key genes which were tightly related to CRPC phenotype and long‐term survival outcomes. Furthermore, Liang et al^39^ investigated core genes for CRPC via bioinformatics analysis, and they found exisulind and phosphodiesterase‐4 inhibitors were potential drugs for CRPC. Consistent with the finding revealed by Song et al,^15^ we also uncovered that the hub genes were associated with PCa progression. Nevertheless, there also existed differences between our present study and theirs. Song et al^15^ identified a module significantly related to Gleason score. In our study, we identified a module that could distinguish CRPC from HSPC and the hub genes were related to PCa prognosis and survival. And we aimed at finding out biomarkers for CRPC and validating their effectiveness.

Co‐expression analysis is a useful technique for multigene analysis of large data sets. Based on these findings, we can apply the hub genes into clinical use in the future. However, some limitations in this study should be taken into consideration. First of all, it is a retrospective research, and data of this research were extracted from public database. Further large‐cohort, multicentre and prospective studies are needed to validate these hub genes that may be related to tumour progression and prognosis. Secondly, this current study is lack of the in vivo experiments of investigating the molecular mechanisms to provide clinical guidance.

## 5 CONCLUSION

Our study picked out and characterized 9 DEGs and significant gene module closely related to CRPC phenotype using WGCNA. Additionally, *HNRNPA2B1*, *GOLGA8B* and *MAPK8IP3* were identified to be tightly associated with tumour progression and prognosis. Further in vivo experiments are needed before clinical application.

## CONFLICT OF INTEREST

The authors declare no conflicts of interests.

## AUTHOR CONTRIBUTION

**Yifei Cheng:** Conceptualization (equal); Data curation (lead); Formal analysis (equal); Software (equal); Writing‐original draft (lead). **Lu Li:** Data curation (equal); Formal analysis (equal); Investigation (equal); Software (lead); Supervision (equal); Writing‐original draft (supporting). **Zongshi Qin:** Formal analysis (equal); Investigation (equal); Methodology (lead); Supervision (equal); Validation (lead); Visualization (equal). **Xiao Li:** Conceptualization (equal); Data curation (equal); Formal analysis (equal); Funding acquisition (lead); Methodology (equal); Project administration (lead); Visualization (equal); Writing‐review & editing (supporting). **Feng Qi:** Conceptualization (equal); Data curation (equal); Formal analysis (equal); Funding acquisition (equal); Investigation (equal); Methodology (equal); Project administration (supporting); Resources (equal); Software (equal); Validation (equal); Writing‐review & editing (lead).

## Supporting information

Figure S1

Figure S2

Figure S3

Figure S4

Figure S5

## Data Availability

The data that support the findings of this study are openly available in the TCGA data set (https://portal.gdc.cancer.gov/) and GEO data set (http://www.ncbi. nlm.nih.gov/geo/).
