# Supplementary figures and images for: Identification of castration‐resistant prostate cancer‐related hub genes using weighted gene co‐expression network analysis
Source: J Cell Mol Med. 2020 Jun 2;24(14):8006–17. doi: 10.1111/jcmm.15432 (PMC7348158; doi:10.1111/jcmm.15432)

Sample dendrogram and trait heatmap

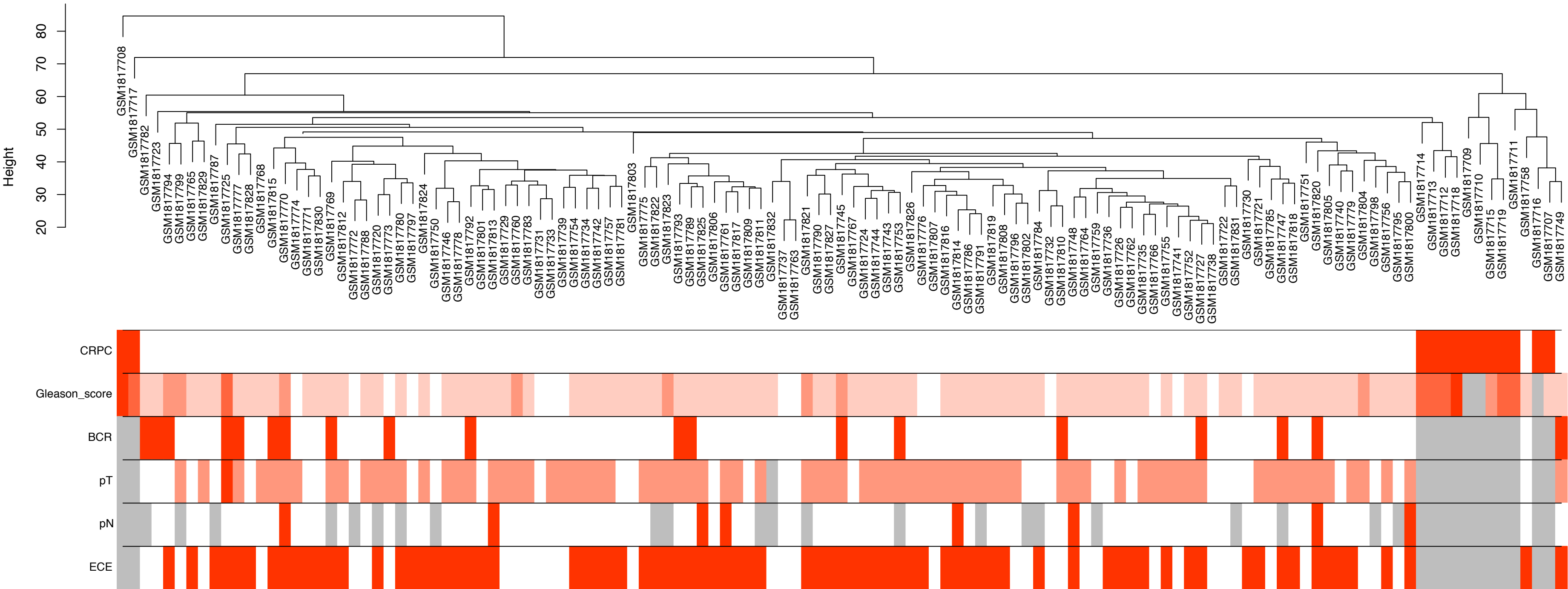

Supplement: Supplementary file 1 — Figure S1 [file JCMM-24-8006-s001.pdf]

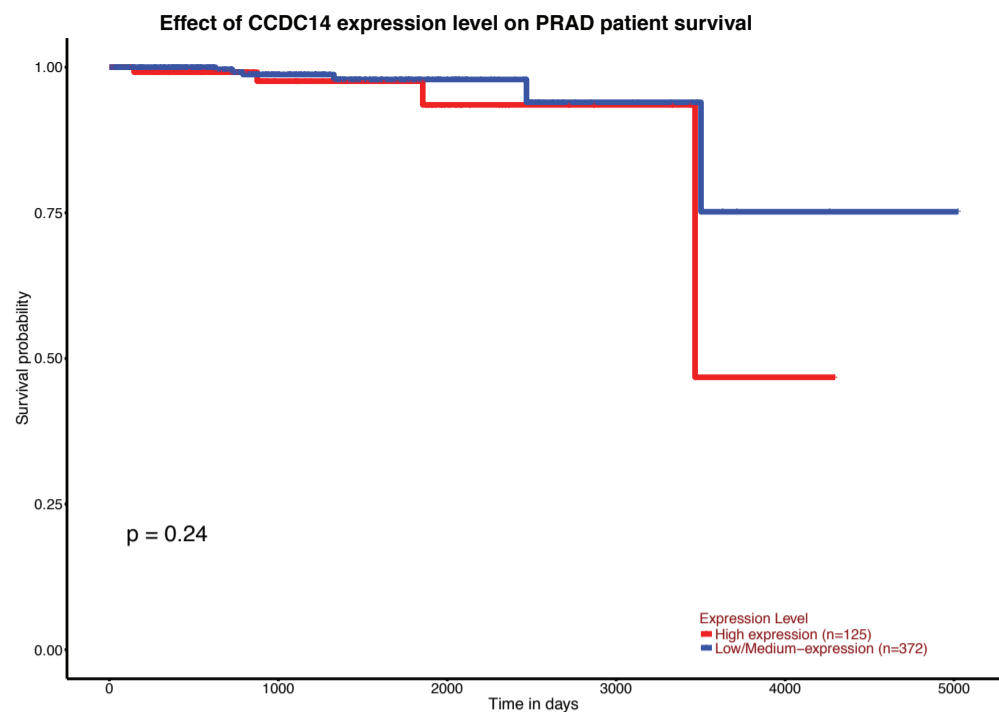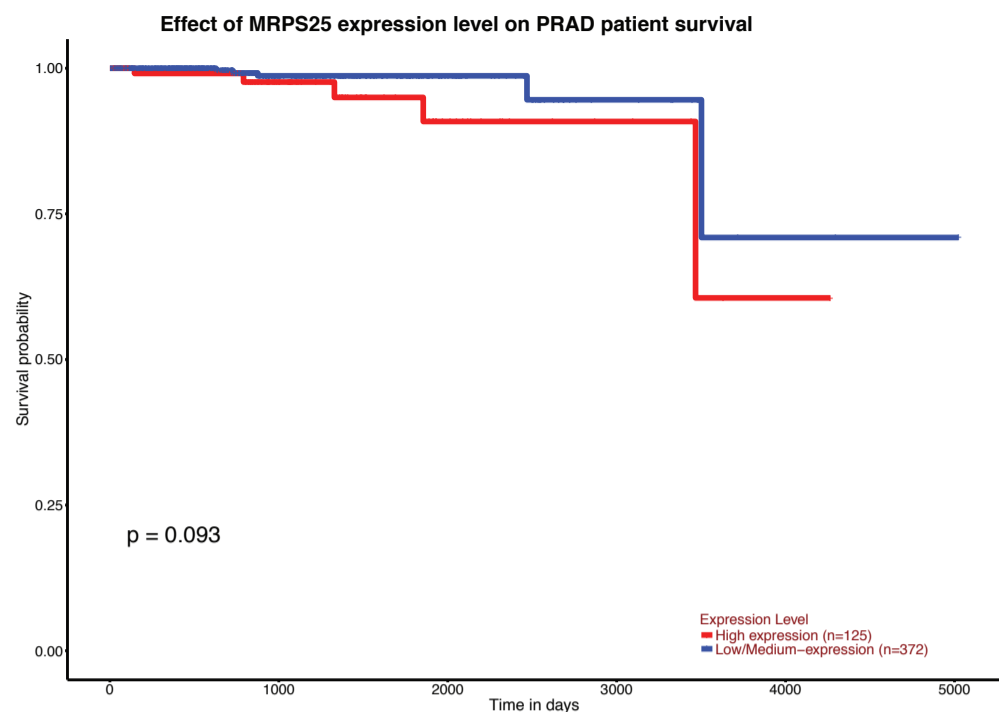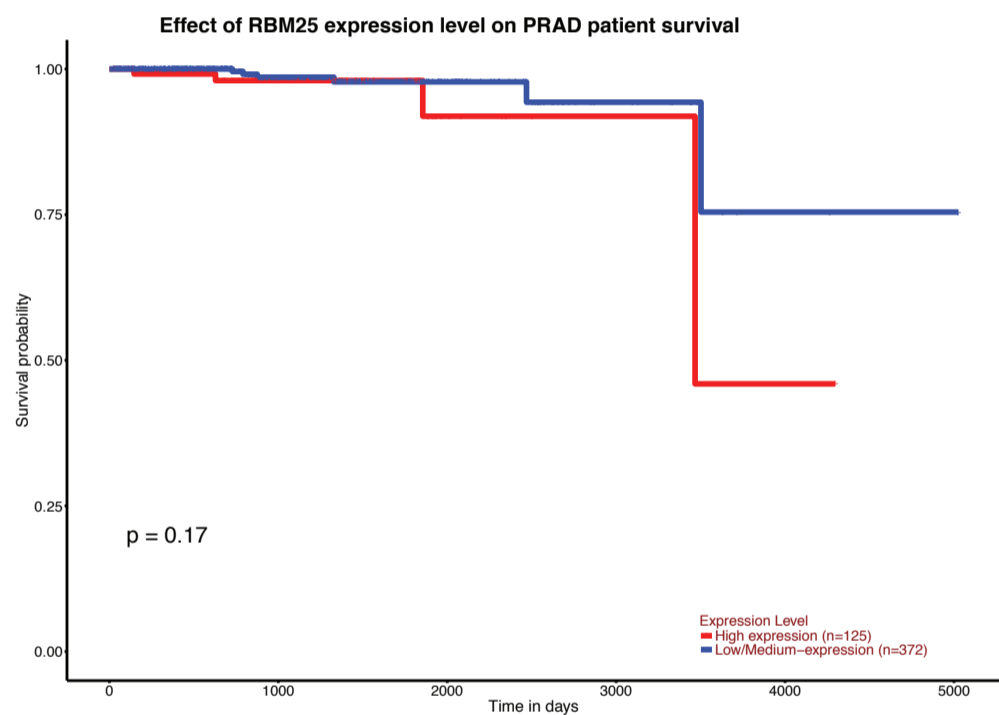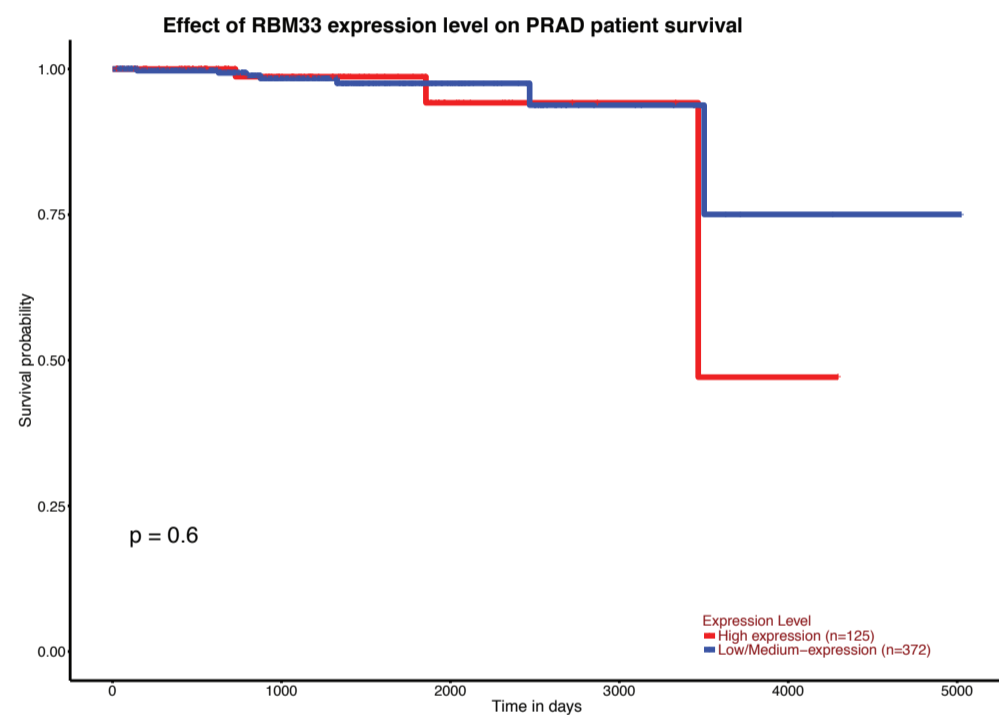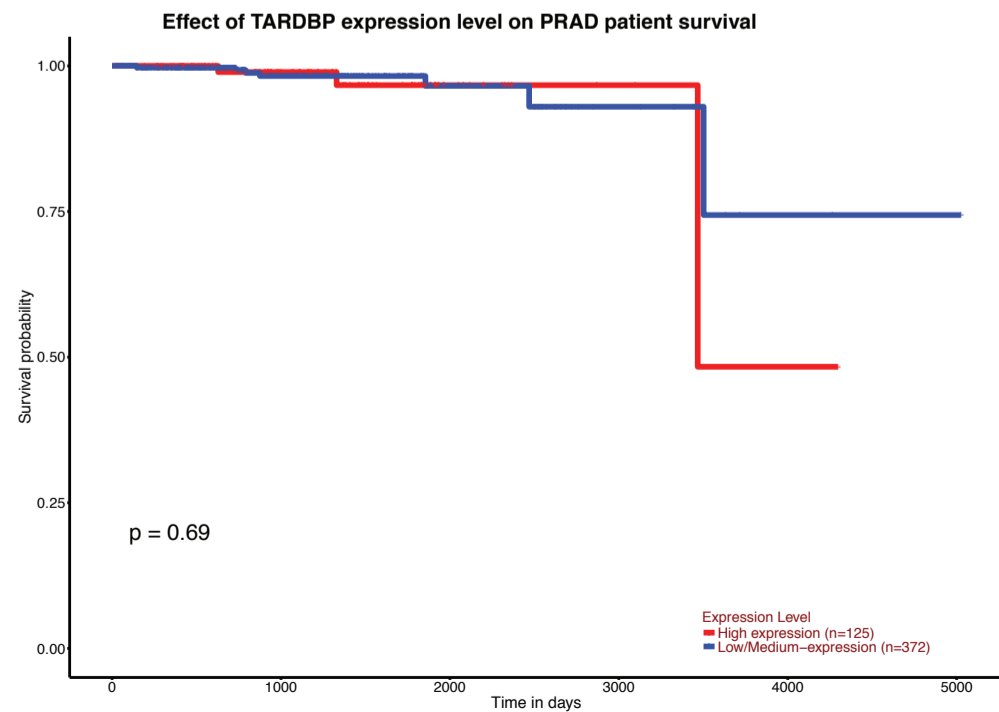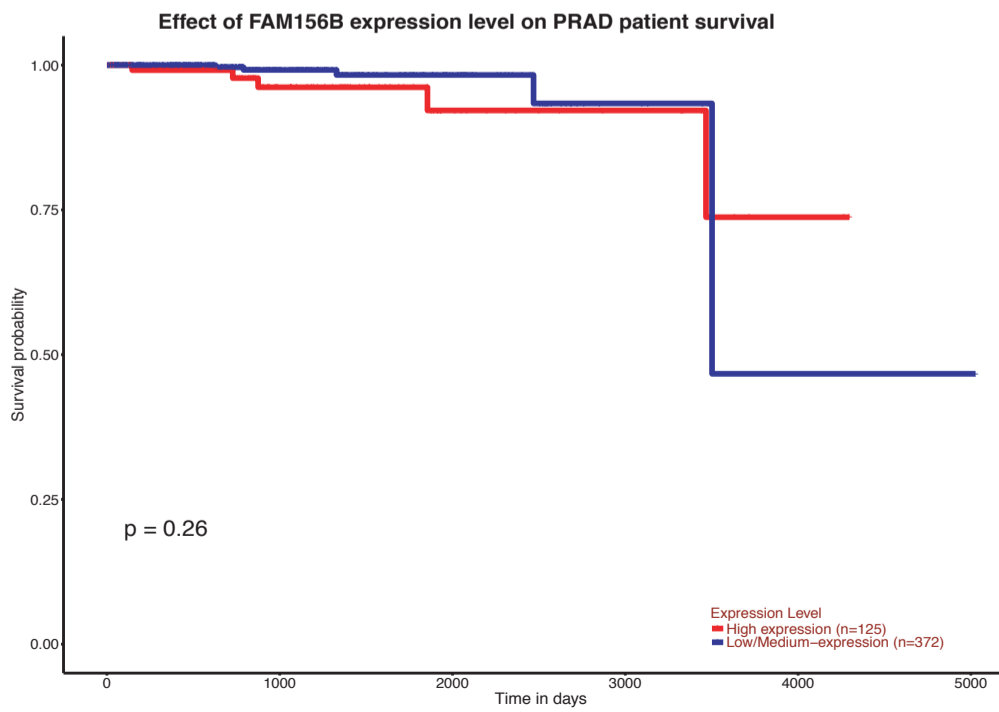

Supplement: Supplementary file 2 — Figure S2 [file JCMM-24-8006-s002.pdf]

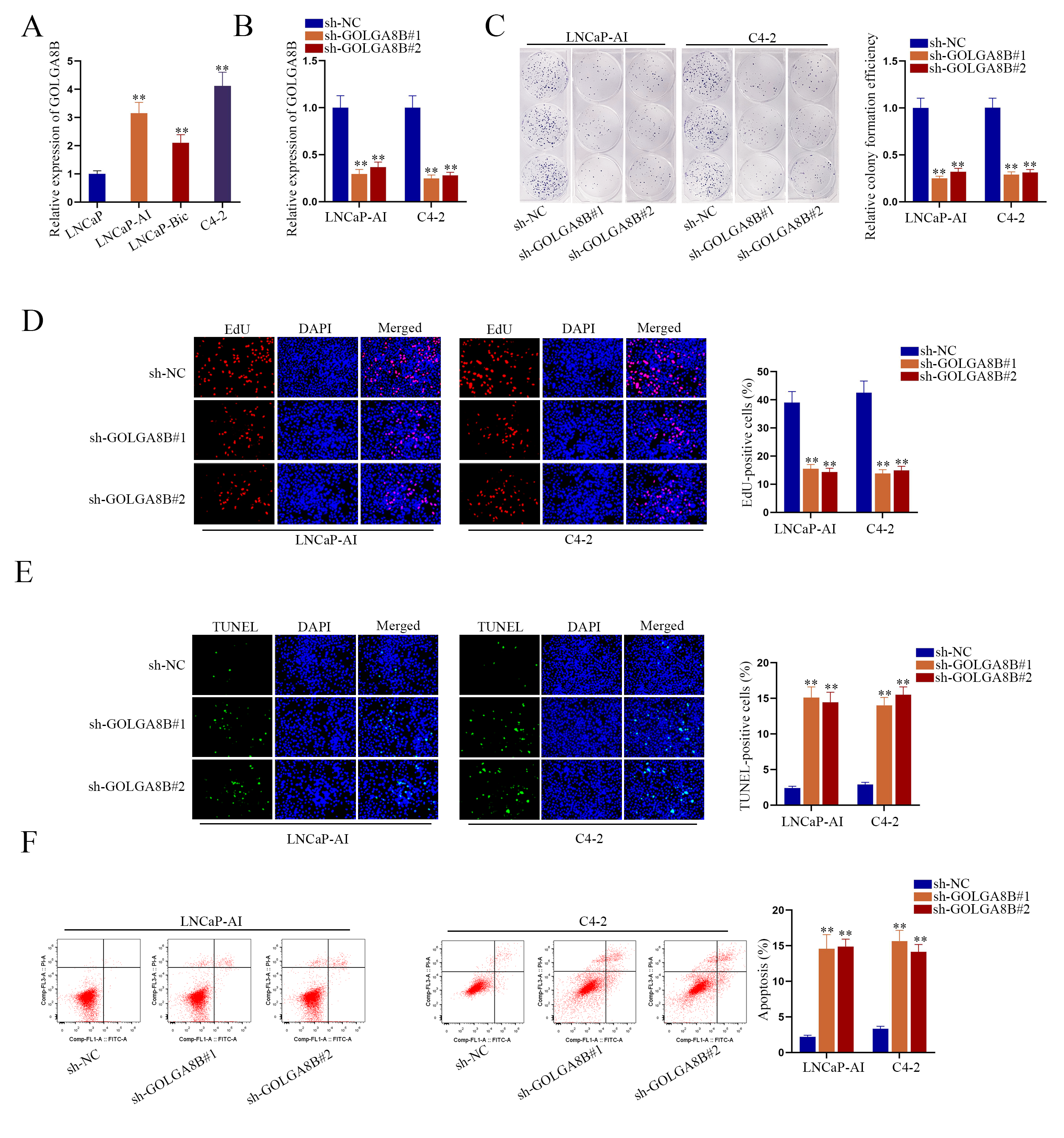

Supplement: Supplementary file 3 — Figure S3 [file JCMM-24-8006-s003.tif]

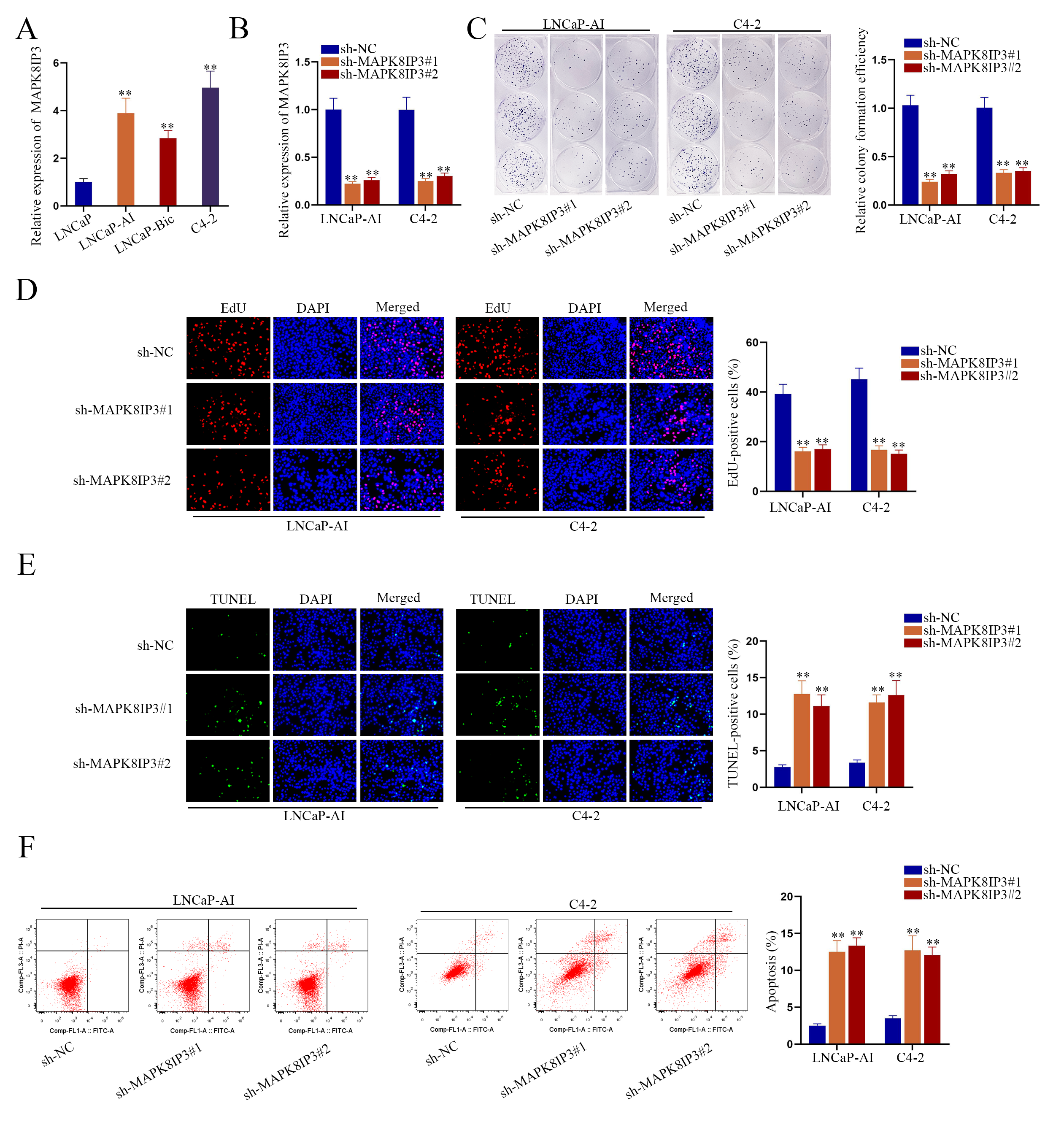

Supplement: Supplementary file 4 — Figure S4 [file JCMM-24-8006-s004.tif]

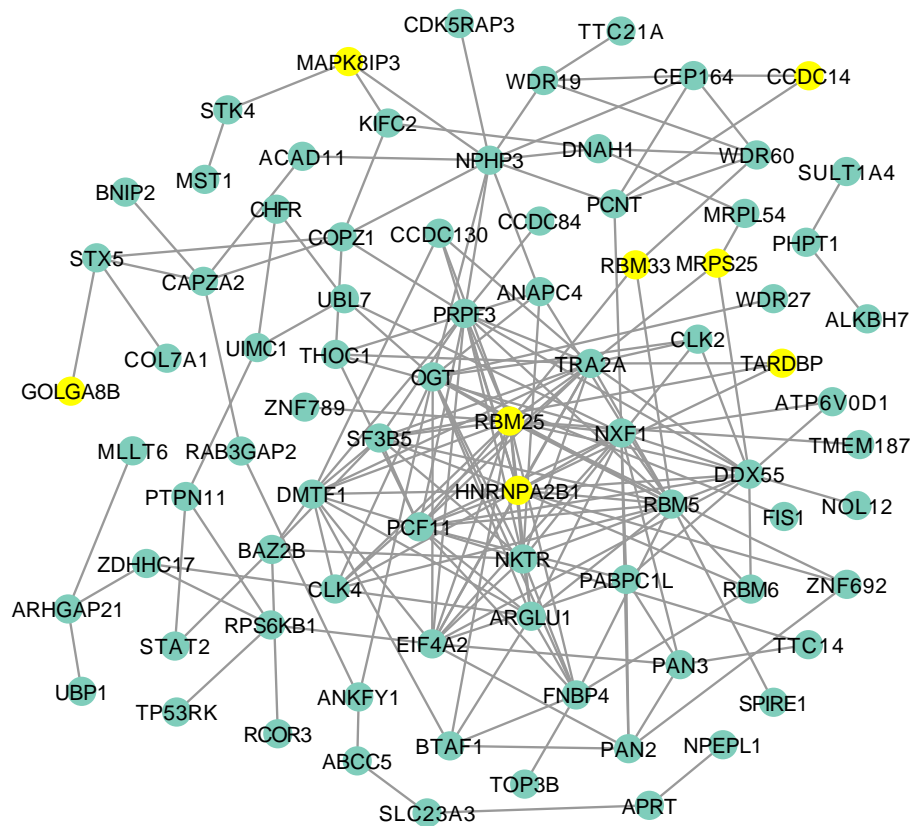

Supplement: Supplementary file 5 — Figure S5 [file JCMM-24-8006-s005.pdf]
